# Exhaustion of Protective Heat Shock Response Induces Significant Tumor Damage by Apoptosis after Modulated Electro-Hyperthermia Treatment of Triple Negative Breast Cancer Isografts in Mice

**DOI:** 10.3390/cancers12092581

**Published:** 2020-09-10

**Authors:** Lea Danics, Csaba András Schvarcz, Pedro Viana, Tamás Vancsik, Tibor Krenács, Zoltán Benyó, Tamás Kaucsár, Péter Hamar

**Affiliations:** 1 Institute of Translational Medicine, Semmelweis University, 1094 Budapest, Hungary; danics.lea@med.semmelweis-univ.hu (L.D.); schvarcz.csaba-andras@med.semmelweis-univ.hu (C.A.S.); pedroleroybio@gmail.com (P.V.); vancsik.tamas@med.semmelweis-univ.hu (T.V.); benyo.zoltan@med.semmelweis-univ.hu (Z.B.); kaucsar.tamas@med.semmelweis-univ.hu (T.K.); 2 1st Department of Pathology and Experimental Cancer Research, Semmelweis University, 1085 Budapest, Hungary; krenacs.tibor@med.semmelweis-univ.hu

**Keywords:** modulated electro-hyperthermia (mEHT), triple-negative breast cancer (TNBC), isogenic mouse cancer, heat-shock protein-70, BALB/C mouse

## Abstract

**Simple Summary:**

Breast cancer is one of the most frequent cancer types among women worldwide. Triple-negative breast cancer is a highly aggressive breast cancer type with very poor survival due to the lack of targeted therapy. Modulated electro-hyperthermia (mEHT) is a newly emerging form of adjuvant, electromagnetic cancer-treatment. Capacitive energy delivery and frequency modulation enable the application of non-thermal effects. Furthermore, selective energy absorption by the tumor (as demonstrated in our present paper) enables 2.5 °C selective heating of the tumor. In the present study, we demonstrate in an in vivo syngeneic Balb/c TNBC mouse model that mEHT caused a remarkable reduction in the number of viable tumor cells accompanied by significant cleaved caspase-3-related apoptotic tumor tissue destruction and a transitional heat shock response. Furthermore, we demonstrated in vitro that the tumor cell killing effect of mEHT was amplified by inhibitors of the protective heat shock response such as Quercetin and KRIBB11.

**Abstract:**

Modulated electro-hyperthermia (mEHT) is a complementary antitumor therapy applying capacitive radiofrequency at 13.56 MHz. Here we tested the efficiency of mEHT treatment in a BALB/c mouse isograft model using the firefly luciferase-transfected triple-negative breast cancer cell line, 4T1. Tumors inoculated orthotopically were treated twice using a novel ergonomic pole electrode and an improved mEHT device (LabEHY 200) at 0.7 ± 0.3 W for 30 min. Tumors were treated one, two, or three times every 48 h. Tumor growth was followed by IVIS, caliper, and ultrasound. Tumor destruction histology and molecular changes using immunohistochemistry and RT-qPCR were also revealed. In vivo, mEHT treatment transitionally elevated Hsp70 expression in surviving cells indicating heat shock-related cell stress, while IVIS fluorescence showed a significant reduction of viable tumor cell numbers. Treated tumor centers displayed significant microscopic tumor damage with prominent signs of apoptosis, and major upregulation of cleaved/activated caspase-3-positive tumor cells. Serial sampling demonstrated substantial elevation of heat shock (Hsp70) response twelve hours after the treatment which was exhausted by twenty-four hours after treatment. Heat shock inhibitors Quercetin or KRIBB11 could synergistically amplify mEHT-induced tumor apoptosis in vitro. In conclusion, modulated electro-hyperthermia exerted a protective heat shock response as a clear sign of tumor cell stress. Exhaustion of the HSR manifested in caspase-dependent apoptotic tumor cell death and tissue damage of triple-negative breast cancer after mEHT monotherapy. Inhibiting the HSR synergistically increased the effect of mEHT. This finding has great translational potential.

## 1. Introduction

Breast cancer is the most common malignant disease and also the most frequent cause of cancer-related death among women [1]. About 15% of all invasive breast cancers are classified as triple-negative breast cancers (TNBC) [2], a very aggressive form [3]. Lack of estrogen-, progesterone, and human epidermal growth factor receptor 2 (HER2) expression in TNBC excludes targeted therapy with presently available drugs. Thus, (neo)adjuvant or complementary therapies are needed to improve TNBC treatment and survival.

Modulated electro-hyperthermia (mEHT) is a non-invasive, complementary therapeutic option, by which selective tumor cell destruction can be induced by loco-regional radiation with amplitude modulated (AM), 13.56 MHz frequency (radiofrequency) electromagnetic field [4]. The communicated energy is detected as a temperature rise in the tumor. Selective power absorption by cancer cells is based on their higher glycolytic rate, discrepant ionic composition, and altered permittivity compared to normal tissues. The selective power absorption results in tumor-specific tissue damage [5] and tumor cell death.

Mechanisms of mEHT at the cellular level include heat generation, which induces cell stress because of protein unfolding and unspecific aggregation [6,7]. In response to stress-related cellular changes, the heat shock response is initiated with the deliberation of major heat shock proteins (Hsps), such as Hsp70 [4,5]. These chaperones prevent damage caused by cell stress through disaggregating, refolding, or degrading damaged proteins [8]. However, if the protection mechanisms are not able to restore normal cell functions, cell stress results first in cell cycle arrest followed by caspase-dependent apoptosis [6,7].

Modulated electro-hyperthermia has been already applied efficiently as monotherapy in numerous experimental models of cancers such as in human hepatocellular carcinoma (HepG2) [9] and human glioblastoma (U87-MG) xenografts in vivo [10]. The comprehensive study by Krenács’s group revealed that mEHT elevated the expression of damage-associated molecular patterns (DAMPs) in human colon adenocarcinoma (HT29) xenografts indicating major cell stress and protein destruction induced by mEHT [5,11]. Similar findings were published by Besztercei et al. on melanoma isografts (B16F10), where mEHT monotherapy also induced massive DAMP expression, DNA damage, and induction of the apoptotic machinery, besides cell cycle arrest [12].

The effects of mEHT on TNBC has not been studied before, probably due to the lack of an efficient device optimized for mice. The former mEHT device for rodents had a large tissue electrode with insufficient contact with the small tumors in the inguinal groin. The 4T1 cell line is one of the most commonly used isogenic BALB/C TNBC cell lines. Specific features of the 4T1 cell line are the epithelial phenotype, strong invasiveness and high metastatic potential, which makes it a suitable experimental model for human TNBC [13,14,15].

Quercetin is a natural flavonoid, which can be found in the common human diet, but is also available as a dietary supplement [16]. Quercetin inhibits multiple target proteins, including heat shock factor 1 (Hsf1), NF-κB, several kinases, and CYP3A4 [17]. Quercetin has been described as a potent inhibitor of the heat shock response (HSR) through blocking Hsf1 kinase and thus, the hyperphosphorylation of Hsf1 [18]. Consequently, Hsf1 cannot be translocated into the nucleus, which is essential for the induction of heat shock proteins, like Hsp70 [19,20]. Furthermore, hyperphosphorylation of Hsf1 leads to the release of bound heat shock proteins such as Hsp70 [21]. However, as Quercetin inhibits not only HSF1 but also NF-κB and AP-1, the anticancer activity of Quercetin is pleiotropic.

KRIBB11 (N2-(1H-indazole-5-yl)-N6-methyl-3-nitropyridine-2,6-diamine) is a novel synthetic chemical compound described by Yoon et al. in 2011. KRIBB11 abolishes the heat shock-dependent induction of Hsp70 through inhibition of Hsf1 by direct binding to Hsf1. KRIBB11 is the first direct inhibitor of Hsf1 and thus, a more potent and specific inhibitor than Quercetin [22,23].

In the present paper, we demonstrate the upgrading of the rodent modulated electro-hyperthermia device—a necessary step to efficiently treat orthotopic mouse breast cancer. Furthermore, our study demonstrates significant antitumor effects of mEHT in monotherapy already after two repeated treatments on TNBC isografts in immunocompetent BALB/c mice. Tumor cell death was associated with cleaved caspase-3 (cC3) upregulation in the damaged area suggesting apoptotic cell death as a consequence of mEHT. Furthermore, Hsp70 was upregulated in the intact tumor cells surrounding the apoptotic area suggesting a heat shock response induced in surviving tumor cells. In the case of antitumor treatments, proper timing—adjusted to the proliferative cycles of the tumors—is important. Our results suggest that the most extensive tumor cell death occurred twenty-four hours after treatment by which time Hsp70-induced protective mechanisms seemed to be exhausted. We demonstrated in vitro, that inhibition of Hsp70 synergistically enhanced the cell-killing effects of mEHT.

## 2. Results

### 2.1. New LabEHY-200 with Pole Electrode Significantly Improved Treatment Feasibility and Accuracy

**Figure 1:**
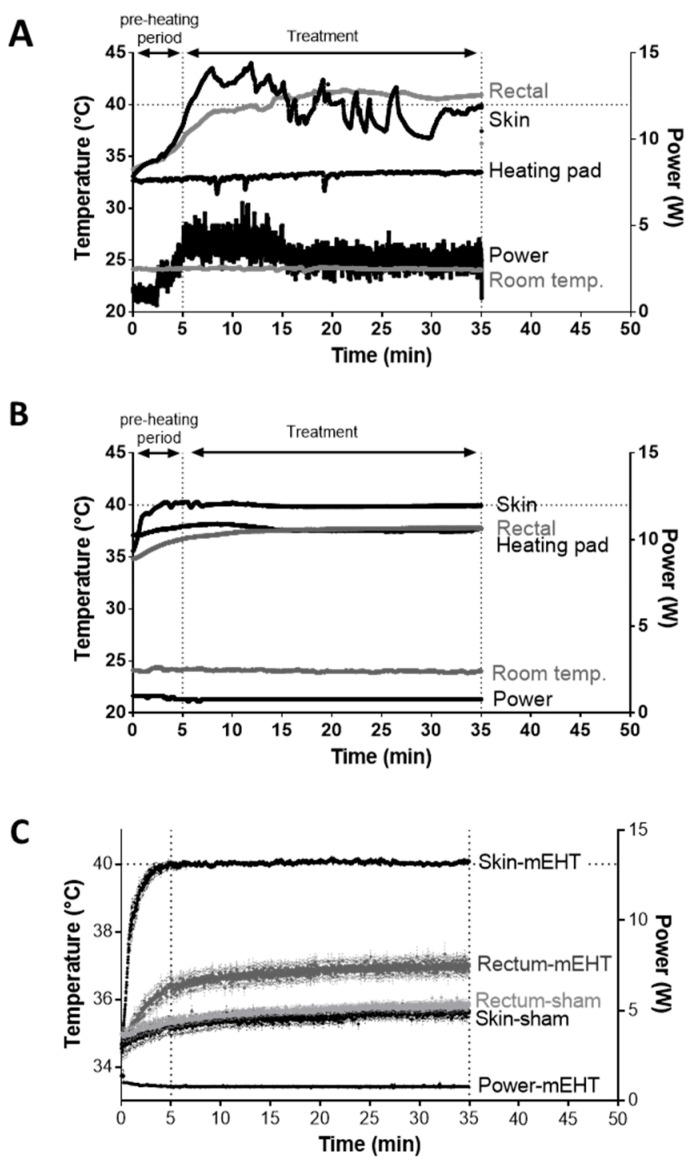
Comparison of LabEHY-100 tissue electrode and LabEHY-200 pole electrode. (**A**) LabEHY-100 with tissue electrode: skin-, rectal-, heating pad- and room-temperatures and applied power during treatment. Representative recording from 1 treatment; (**B**) LabEHY-200 with pole electrode: skin-, rectal-, heating pad-, and room-temperatures and applied power during treatment. Representative recording from 1 treatment; (**C**) temperature recordings from 12 mEHT and 12 sham treatments with LabEHY-200 and pole electrode. *n* = 12/group, Mean ± SEM.

The former LabEHY-100 with tissue electrode (see Methods) was not optimal for treating tumors in the inguinal region of mice as mEHT also elevated the rectal temperature during treatment. Consequently, treatment implementations were problematic, temperature curves were highly variable, more power had to be applied (Figure 1A) and treatments caused heat-related side effects such as skin-burning.

The development of the LabEHY-200 (see Methods) made the treatments more efficient and more standard with lower power (Figure 1B). Together with the newly developed pole electrode (see Methods) a more accurate skin-electrode contact was achieved thus, tissue-coupling improved. The more focused treatments prevented heat-related side-effects. Better focus and coupling reduced the variability in temperature and power during treatments. The rectal temperature remained within the normal range (T_rectal_ = 37.03 ± 0.61 °C) and treatments became local and reproducible (Figure 1B,C).

### 2.2. mEHT Induced Local Temperature Increase in the Tumor

**Figure 2:**
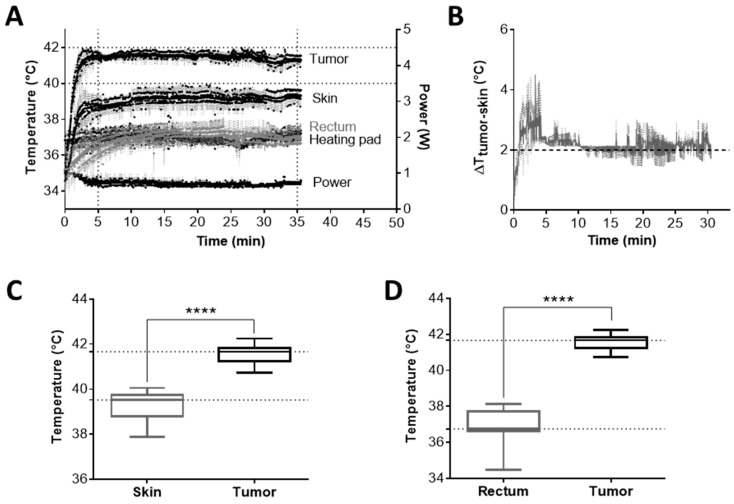
Selective warming of the tumor tissue. (**A**) Temperature curves of the tumor, skin, and rectum during mEHT-treatment (*n* = 5). (**B**) Temperature gradient between the tumor core and skin above the tumor (*n* = 5). Mean ± SEM. (**C**,**D**) Temperature of skin (**C**) or rectum (**D**) vs. tumor core during treatment. Average and box and whiskers: min to max, **** *p* < 0.0001.

To properly adjust the amount of energy delivered into the tumor by mEHT, we determined the temperature within the tumor and its surroundings (skin, rectum) in a pilot experiment (Figure 2A). The tumor temperature was 2.5 ± 0.5 °C higher (*p* < 0.0001) compared to the skin directly above the tumor and 4.4 ± 1.2 °C higher (*p* < 0.0001) than in the rectum below the tumor (Figure 2B). Based on these findings, only the noninvasive rectal and skin temperatures were recorded in all further experiments to monitor the energy communicated to the tumor without any mechanical injury.

### 2.3. mEHT Decreased the Number of Viable Tumor Cells as Detected by In Vivo Fluorescence but did not Influence Tumor Size after Only Two Treatments

**Figure 3:**
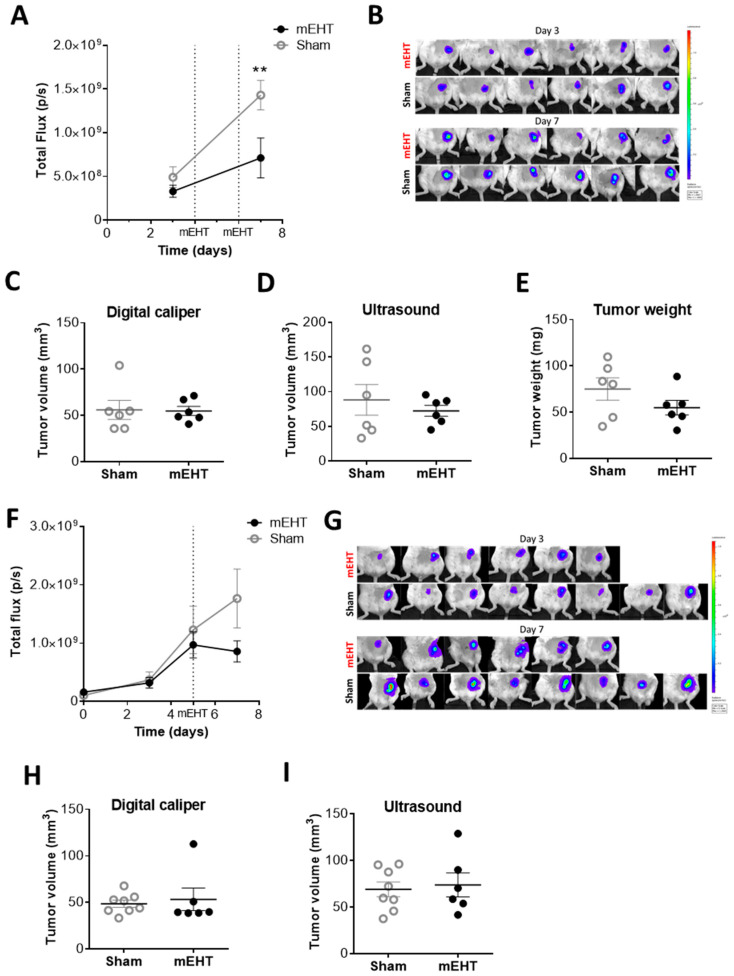
The effects of modulated electro-hyperthermia (mEHT) on tumor size. (**A**,**B**) Total fluorescent flux measured by IVIS 24 h before and after two mEHT treatments; (**C**–**E**) Tumor volumes after two mEHT treatments, measured by a digital caliper (**C**) ultrasound (**D**) and tumor weight after removal (**E**) (*n* = 6/group); (**F**,**G**) Total fluorescent flux measured by IVIS before and after one mEHT treatment, at day 0, 3, 5, and 7; (**H**,**I**) Tumor volume after one mEHT treatment, measured by a digital caliper (**H**) and ultrasound (**I**). (*n*_sham_ = 4, *n*_mEHT_ = 6. Mean ± SEM; (**A**,**F**) two-way ANOVA, Bonferroni correction, ** *p* < 0.01; (**C**–**E**,**H**,**I**) Mann–Whitney test.

Luciferase transfected 4T1 cells emitting fluorescent light were significantly reduced already after two consecutive mEHT treatments (*p* = 0.0082) (Figure 3A,B). However, two treatments did not reduce tumor size as measured by digital caliper and ultrasound (Figure 3C,D). Neither was the tumor weight reduced at termination 24 h after the second mEHT treatment (Figure 3E). We observed a similar tendency after one treatment. The fluorescent light emitted by viable tumor cells was tendentially but not significantly lower in mEHT-treated tumors (Figure 3F,G). Tumor size measured by digital caliper and ultrasound did not differ between the groups after one treatment (Figure 3H,I).

### 2.4. mEHT Treatment Induced Tumor Destruction

**Figure 4:**
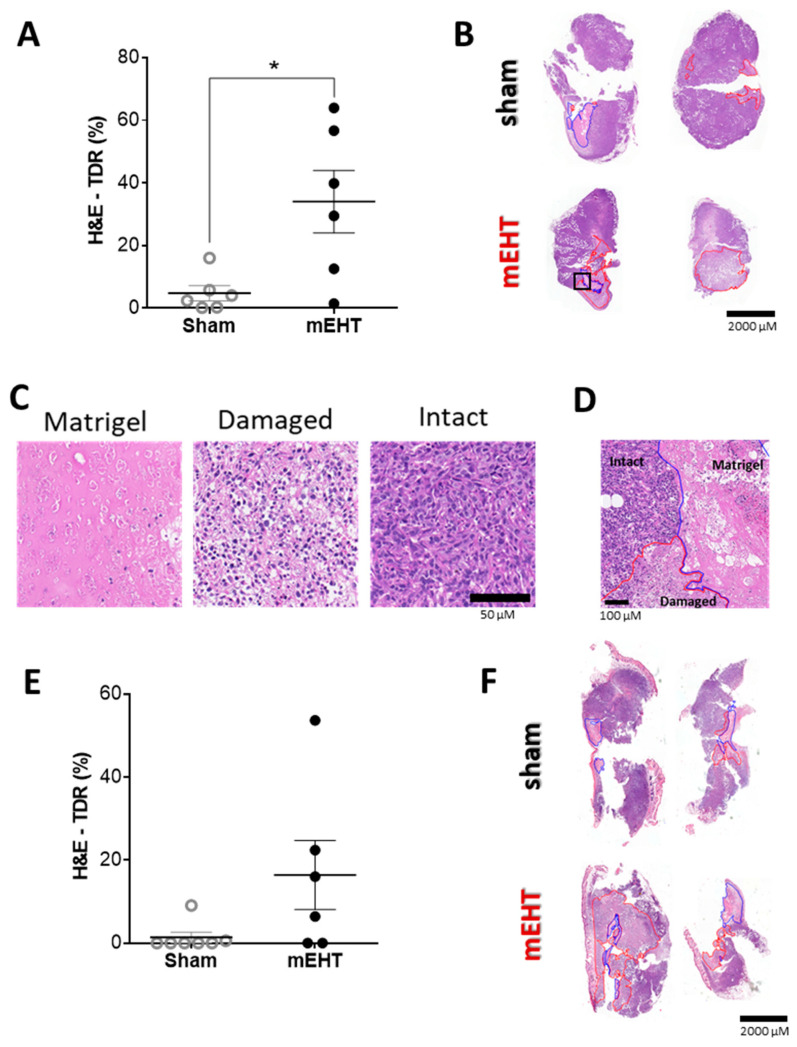
mEHT-induced tumor destruction. (**A**,**B**) Tumor destruction 24 h after two mEHT treatments. (**A**) Tumor destruction ratio (TDR) evaluated on hematoxylin-eosin (H&E) stained sections; (**B**) Apoptotic areas (red contour) and Matrigel^®^ (blue contour) on representative H&E stained sections; (**C**) Matrigel^®^ and damaged tumor areas on H&E stained sections (Magnification: 36×); (**D**) representative image of intact vs. damaged area and Matrigel^®^ (magnified from the H&E section: B/black rectangle, magnification: 8.5×); (**E**,**F**) Tumor destruction 24 h after one mEHT treatment; (**E**) TDR evaluated on H&E stainings; (**F**) Apoptotic areas (red contour) and Matrigel (blue contour) on representative H&E stained sections. Mean ± SEM, Mann–Whitney test, *n* = 6/group, * *p* = 0.0174.

The reduced number of vital tumor cells—as detected by IVIS—was corroborated by the histologic analysis. Following two mEHT treatments, the Tumor Destruction Ratio (TDR) was significantly higher in all mEHT-treated tumors than in the sham-treated ones on the hematoxylin and eosin (H&E) stained sections (*p* = 0.0174) (Figure 4A,B). One single treatment also caused tumor destruction in some of the treated tumors—preferentially in the larger ones, but some tumors were not affected (Figure 4E,F).

### 2.5. Repeated mEHT Induced Hypocellularity of Treated Tumors

**Figure 5:**
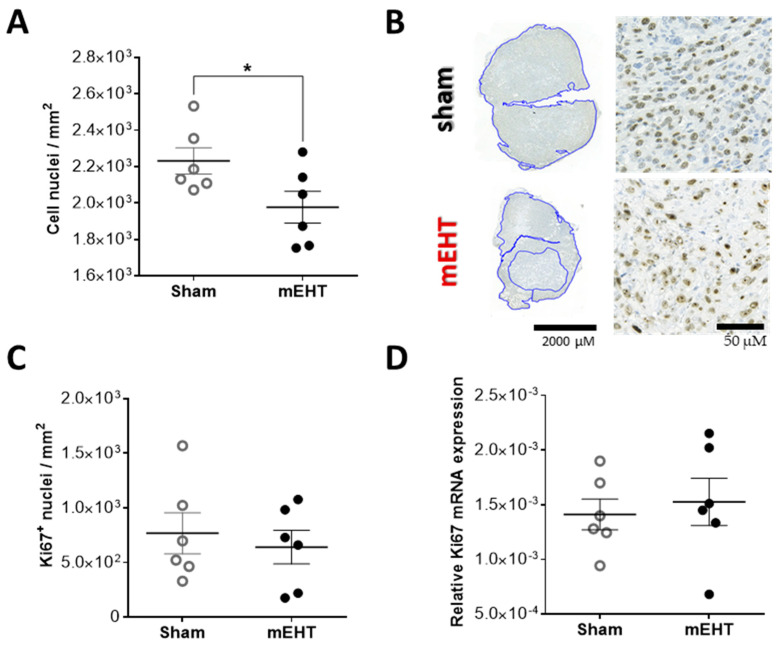
Ki67 expression 24 h after two mEHT treatments. (**A**) Number of all cell nuclei in the intact tumor area; (**B**) Ki67 staining in the viable tumor tissue (blue assigned areas), representative sections; (**C**) Number of strong Ki67-positive nuclei; (**D**) Ki67 mRNA expression in tumor tissue (normalized to 18 S rRNA). Mean ± SEM, unpaired *t*-test, *n* = 6/group, * *p* = 0.0494.

The total number of cell nuclei—evaluated on 3,3’-diaminobenzidine (DAB) stained sections—in the mEHT-treated tumors was significantly smaller than in the sham-treated tumors, resulting in a lower density of viable tumor cells in the mEHT treated tumors, supporting the observation of less viable tumor cells with IVIS after two mEHT treatments (Figure 5A,B). The number of Ki67-positive nuclei evaluated in the intact tumor area (Figure 5C), as well as Ki67 mRNA (Figure 5D), was similar between the groups.

### 2.6. mEHT-Treatment Induced Caspase-Dependent Apoptosis

**Figure 6:**
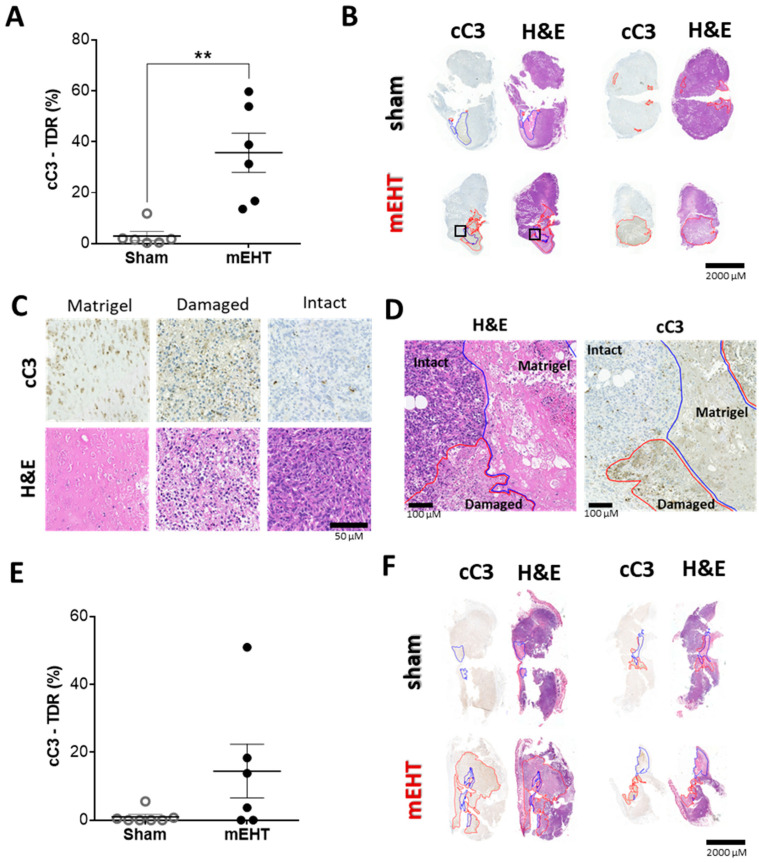
mEHT-induced tumor destruction was cleaved caspase-3 mediated. (**A**,**B**) Tumor destruction 24 h after two mEHT treatments. (**A**) Tissue destruction ratio (TDR) evaluated in cleaved caspase-3 (cC3) stained sections; (**B**) Apoptotic areas (red contour) and Matrigel^®^ (blue contour) on representative cC3-stained sections and consecutive H&E stained sections. (**C**) Matrigel^®^ and damaged tumor areas on cC3 and H&E stained sections (Magnification: 36×); (**D**) Representative images of intact vs. damaged area and Matrigel^®^ (magnified from H&E and cC3 sections: B/black rectangles, magnification: 8.5×); (**E**,**F**) Tumor destruction 24 h after one mEHT treatment; (**E**) TDR evaluated on cC3 stainings; (**F**) Apoptotic areas (red contour) and Matrigel (blue contour) on representative cC3-stained sections and consecutive H&E-stained sections. Mean ± SEM, Mann–Whitney test, *n* = 6/group, ** *p* = 0.0020.

Analysis of cleaved caspase-3 (cC3) immunohistochemical staining of resected tumors demonstrated a significant increase (** *p* = 0.0020) in the cC3-positive area after 2 mEHT treatments compared to the sham tumors (Figure 6A), which extensively overlapped with the damaged areas seen on consecutive H&E sections (Figure 6B–D). We observed a similar tendency already after one mEHT treatment, however, the increase in the cC3-positive damaged area was not significant (Figure 6E,F).

### 2.7. Repeated mEHT Induced Heat Shock Protein 70 (Hsp70) in Viable Tumor Cells

**Figure 7:**
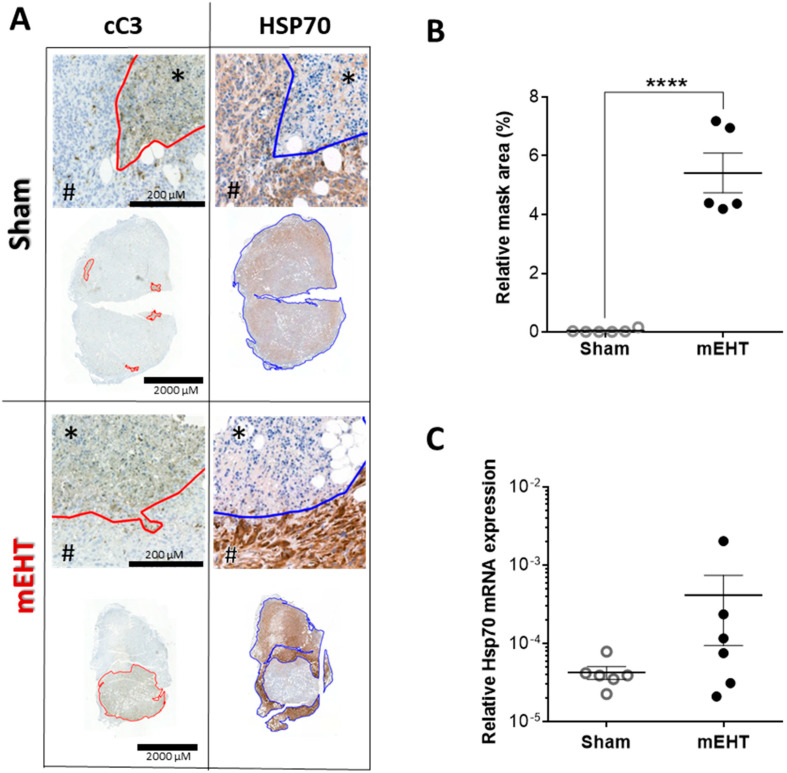
Heat shock protein 70 (Hsp70) expression in tumor tissue 24 h after two mEHT treatments. (**A**) Representative cC3- and Hsp70-stained sections with high and low magnification. The damaged area is cC3-positive (marked with *). The living area is cC3-negative (marked with #). Red line: the border between the damaged and living area. The Hsp70 expression was measured in the living area (marked with *). Blue line: borders of the living area; (**B**) Relative Hsp70-stained mask area of the viable tumor tissue; (**C**) Hsp70 mRNA expression (normalized to 18S rRNA); Mean ± SEM, Mann-Whitney test, *n* = 6/group, **** *p* < 0.0001.

Twenty-four hours after two mEHT treatments the tumors had intense Hsp70 (specific: dark brown/DAB/) staining of viable tumor cells surrounding the damaged tumor area. Similar intense staining was absent in sham-treated tumors (*p* = 0.0028; Figure 7A,B). At this timepoint, Hsp70 mRNA was also elevated in some of the mEHT-treated tumors but not in all of them (Figure 7C).

**Figure 8:**
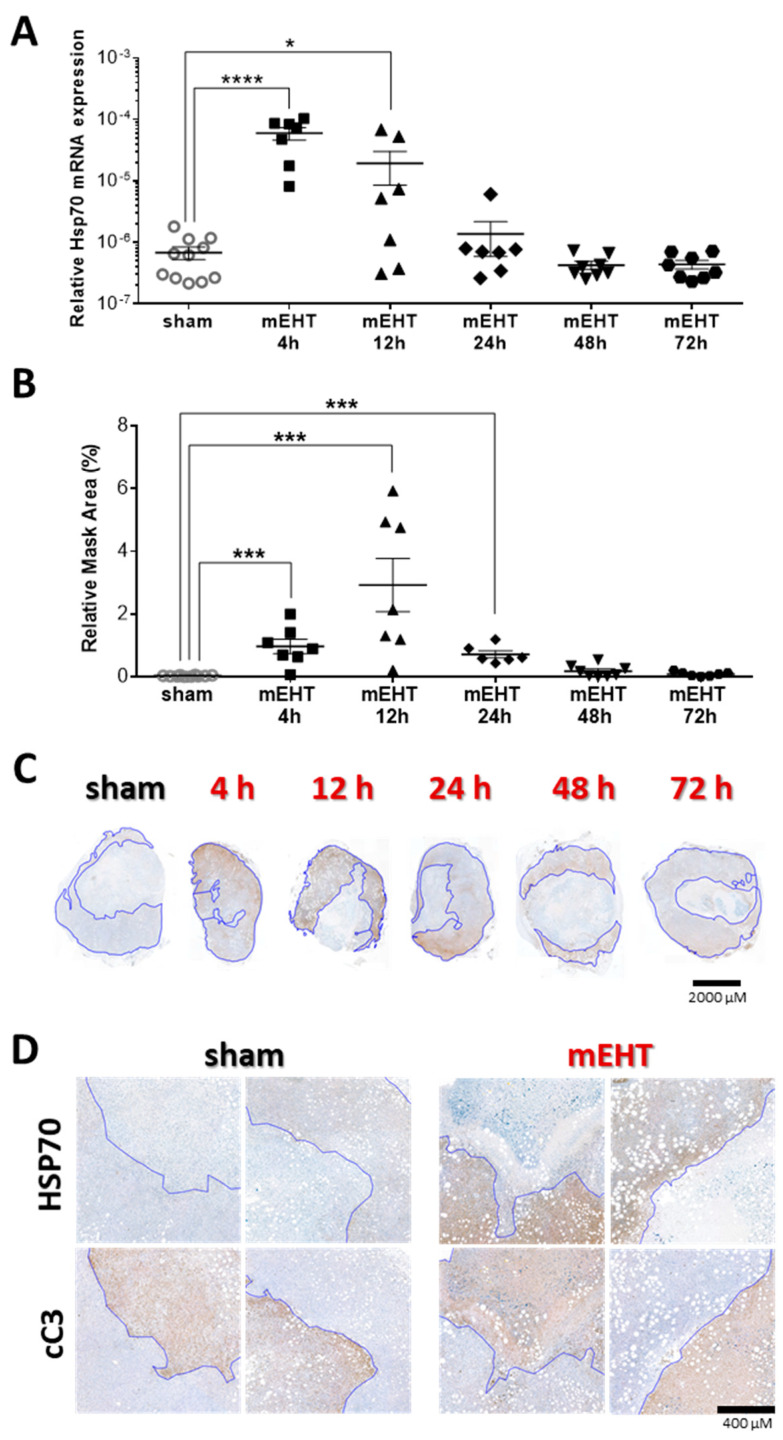
Time kinetics of Hsp70 expression after three mEHT treatments. (**A**) Hsp70 mRNA expression of the tumors at different time-points after treatment; (**B**) Area-proportional expression of Hsp70 protein at different time points after treatment; (**C**) Representative tumor images from the sham and mEHT groups; (**D**) High-magnification images of cC3 and Hsp70 stainings from sham and mEHT treated tumors. Blue line marks the border between live and damaged tumor area assigned based on the cC3-stainings, (Magnification: 5×); (**A**,**B**) sham vs. mEHT, unpaired Mann–Whitney test. Mean ± SEM, * *p* = 0.03, *** *p* < 0.001, **** *p* < 0.0001.

According to the time-kinetics experiment, Hsp70 mRNA peaked already at 4 h post-treatment and started to decline already at 12 h in most tumors (Figure 8A). At 24 h Hsp70 mRNA was already at the sham level in all, except one, tumor and decreased further in the 48- and 72-h post-treatment groups (Figure 8A). On the IHC sections, there was a significant elevation of Hsp70 protein already at 4 h post-treatment, and the peak was at 12 h closely following Hsp70 mRNA (Figure 8A,B). Hsp70 protein started to decrease at 24 h (compared to the 12 h group), but it was still significantly elevated compared to the sham group (Figure 8B–D). Hsp70 protein was at the sham level in all tumors 48 h post-treatment similarly to Hsp70 mRNA (Figure 8).

### 2.8. Tumor-Destructive Effect of mEHT Was Time-Dependent

**Figure 9:**
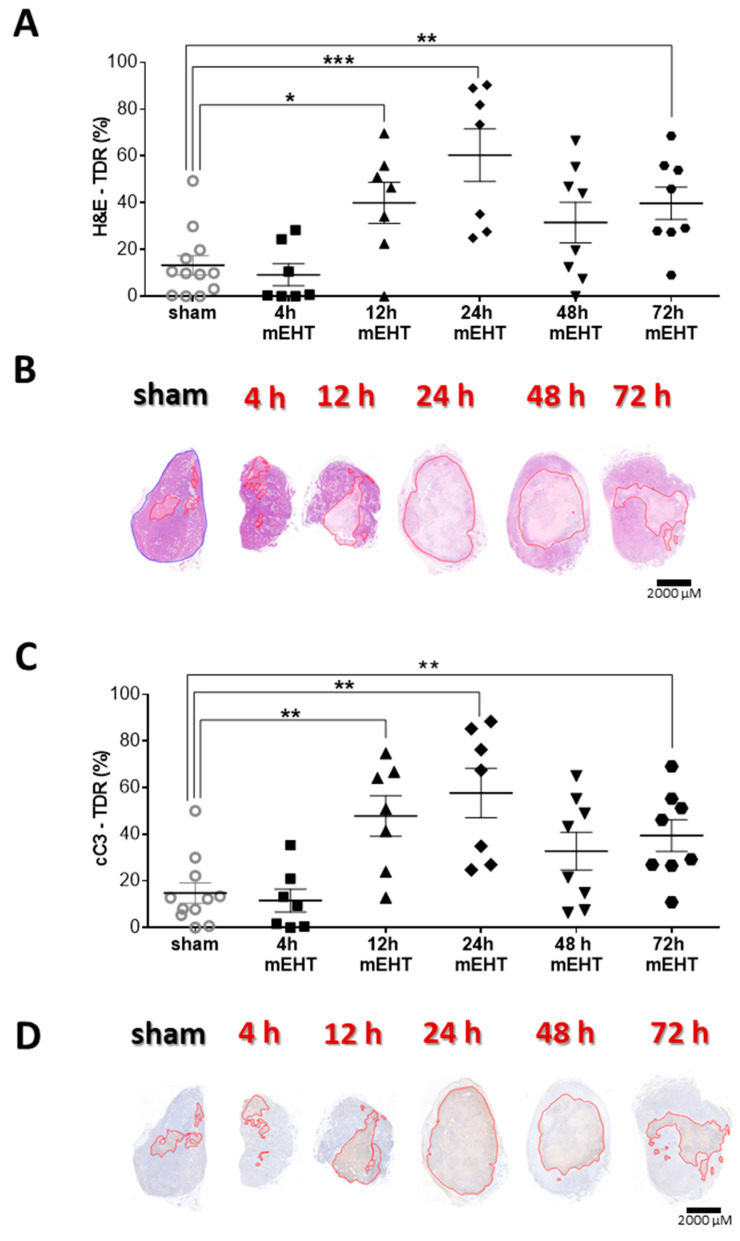
Time kinetics of tumor tissue destruction after mEHT. (**A**) Quantification of tumor destruction ratio (TDR %) on hematoxylin-eosin (H&E)-stained tumors; (**B**) Representative images of H&E-stained sections from sham and mEHT-treated tumors from 4, 12, 24, 48, and 72 h after 3 treatments. Red lines mark the damaged area; (**C**) Quantification of TDR % on cC3 stained sham and mEHT treated tumors; (**D**) Representative images of cC3-stained sections from sham- and mEHT-treated tumors. Red lines mark the damaged area; (**A**,**C**) unpaired Mann–Whitney test. Mean ± SEM, *n* = 6–12/group, * *p* < 0.05, ** *p* < 0.01, *** *p* < 0.001.

Examining the temporal changes in mEHT effectiveness, TDR (%) on H&E and cC3 immunohistological stainings were evaluated at different time points after three mEHT treatments. TDR (%) reached it’s peak 24 h after the third treatment, compared to sham, suggesting, that the protective mechanisms, marked by Hsp70 expression, had run down by this time (Figure 9A,B). The proportion of the cC3-positive area increase in the treated tumors, 24 h after the third treatment and overlapped with the damaged areas on the H&E sections (Figure 9C,D).

### 2.9. Tumor-Cell-Killing Effect of mEHT Was Enhanced Synergistically by Combination with Heat Shock Inhibitors

**Figure 10:**
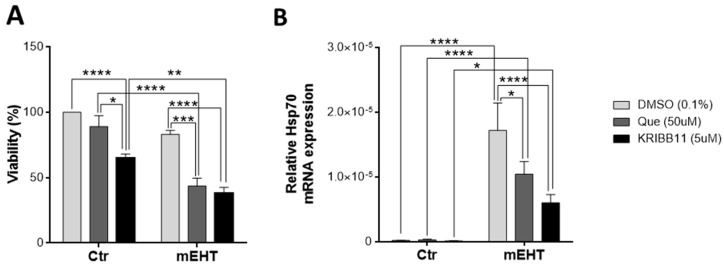
Synergistic effect of mEHT and heat shock inhibitors. 4T1 cells pre-treated with heat shock response inhibitors, Quercetin or KRIBB11, or vehiculum (DMSO) for 1 h before mEHT (42 °C, 30 min) treatment. (**A**) Resazurin viability assay, 24 h post-mEHT, viability expressed as percent of control (37 °C, vehiculum only). (**B**) Hsp70 mRNA 2 h post- mEHT, normalized to 18S rRNA. Two-way ANOVA, Mean ± SEM, *n* = 5–12/group, * *p* < 0.05, ** *p* < 0.01, *** *p* < 0.001, **** *p* < 0.0001.

In vitro treatment of 4T1 cells with two potent heat shock response inhibitors: Quercetin and KRIBB11, resulted in lower tumor cell viability 24 h post-treatment. Only KRIBB11 was able to reduce viability significantly in monotherapy. Combined treatment with mEHT resulted in a synergistic effect as both combinations lead to lower viability than the sum of the viability losses due to the two single therapies applied individually (Figure 10A). MEHT treatment upregulated Hsp70 mRNA 2 h post-treatment. Both Quercetin and KRIBB11 significantly inhibited Hsp70 mRNA upregulation compared to the mEHT + vehiculum (DMSO) group (Figure 10B).

## 3. Discussion

In this study we applied for the first time the new LabEHY-200 device with an ergonomically designed pole electrode, improved and optimized for mouse breast cancer treatment. This new setup allowed highly selective tumor treatment as detected by selective warming of the tumor tissue. The core temperature of the tumors was 2.5 ± 0.5 °C higher compared to the skin temperature above the tumor. The temperature gradient was reproducible and constant during treatments. Others reported that under similar treatment conditions the temperature difference was comparable between the core of the tumor and the surface of the skin above the tumor (ΔT_(tumor-skin)_ ≥ 2 °C) in other tumor types [9,12,24,25,26]. However, this selective increase of tumor temperature is a specific feature of modulated electro-hyperthermia, since with whole-body hyperthermia or non-modulated radiofrequency capacitive hyperthermia approaches heat accumulation due to hypovascularization of tumors generally does not allow ΔT > 1 °C, and heating is inhomogeneous [27,28]. The energy trap hypothesis in our case is supported by the observation of tumor destruction in the cores of the tumors. Furthermore, an important reason for the highly effective and selective heating and killing of the tumor by mEHT is amplitude-modulation (AM) and its non-thermal effects on the tumor cells. The non-thermal effects, in addition to the thermal effect of the dielectric heating by the 13.56 MHz carrier frequency, resulting in much larger tumor destruction than expected from non-modulated radiofrequency capacitive hyperthermia [29,30,31]. The non-thermal effects are also demonstrated in cell culture experiments, where mEHT treatment is compared with conventional warming to similar temperatures [29,32].

In our experiments, the specific absorption rate (SAR) achieved in the tumors was around 90 W/kg, which is higher than the usual SAR (30–40 W/kg) in clinical use [33]. However, in some cases, documented by Griffiths et al., SAR values even as high as 89 W/kg—which is comparable to the SAR in our experiment—could be achieved during human hyperthermia treatments, mostly in superficial tumors [34]. The bigger size of human tumors may also contribute to explaining why SAR values are lower in clinical practice than in our mouse experiment, as larger tumors need lower SAR to achieve the same temperature. Nevertheless, we also have to admit, that, in the case of modulated electro-hyperthermia the standard way of SAR calculation (see formula below) is misleading as during mEHT the energy is absorbed by lipid rafts of the tumor cell membranes (nanoheating) and this leads to the warming up of the whole tumor [31,35]. Thus, the actual weight of the heated target (lipid rafts) is much lower than the weight of the tumor. The estimated value of SAR is ~1 kW/kg, which corresponds to nanoparticle heating [36]), and the heating is heterogeneous. As SAR calculation is dependent on the weight of the heated target as well as on the average temperature of the target, its usefulness for the definition of thermal parameters of the treatment is limited [31].

SAR = ΔT × C/t

(ΔT: temperature gradient, C: specific heat of the biological material [J/K·kg], t: time [s]).

During mEHT treatment, the energy absorption in the tumor causes cell stress and initiates the expression of cell stress-associated molecules [37]. One of the most abundant stress-induced molecules is Hsp70, which becomes strongly expressed in response to heat stress and damage. [8] Hsp70 has ambivalent effects on tumor progression as it can mediate both tumor-promoting and tumor-suppressing pathways, depending on its macroscopic (tumor cells or tumor stroma) and microscopic (intracellular or extracellular) localization [38,39]. Juhasz et al. provide a detailed description of the tumor-suppressing effects of extracellular Hsp70, which acts as a danger signal and induces the activation of the immune system against the tumor cells [38]. As in our experiments, 4T1 tumors had very poor immunogenicity and very low rate of tumor-infiltrating immune cells [40] we assumed, that the immune cells-mediated tumor-suppressing effects of Hsp70 may be negligible in the 4T1 model. This was confirmed by the fact, that inhibiting Hsp70 in vitro significantly augmented the tumor-destructive effect of mEHT (discussed in detail later). It has been reported that mEHT provoked Hsp70 in HepG2 hepatic [41], B16F10 melanoma [12], and CT26 [42,43] colorectal cancer cells after mEHT treatment, in vitro and in B16F10 [12], CT26 [4], and HT29 [11] tumors, in vivo. Similarly, we also found that mEHT treatments induced substantial damage in the core of the TNBC tumors surrounded by extensive Hsp70 expression. The central damage and the Hsp70 positive “ring” around the dead area supports the assumption that mEHT heated the tumor centrally, and that the tumor destruction began in the core of the tumor and progressed outward. The cells surrounding the damaged area must have been exposed to severe stress by mEHT, but the high Hsp70 expression protected them from cell death. The lack of similarly strong staining in sham-treated tumors demonstrates the specific effect of mEHT. The time kinetic experiment demonstrated, that Hsp70 mRNA peaked already 4 h post-treatment, followed by a peak of Hsp70 protein at 12 h. Andócs at al. also reported the same dynamics of Hsp70 mRNA changes after mEHT treatment [11]. As Hsp70 protein returned close to sham level at 24 h we observed tumor cell death marked by cleaved caspase-3 positivity, suggesting that exhaustion of Hsp70-mediated protection led to apoptotic cell death. The mEHT-induced tumor cell destruction is a long-lasting effect, as TDR was still significantly higher in mEHT-treated tumors 72 h post-treatment compared to sham. Previous reports support that mEHT induces cC3 activation in vitro in human ovarian (OVCAR-3), cervical (SNU-17) [25], and mouse colorectal (CT26) [4] carcinoma cells. The cleaved caspase-3 expression also increased in CT26 cells in vivo as a result of mEHT monotherapy [4,42]. However, here we present for the first time, that mEHT first activates protective machinery (e.g., Hsp70 induction), than in consequence of the exhaustion of these protective mechanisms cells undergo caspase-3-mediated cell death. Although we cannot exclude necrotic cell death, the cC3 positivity of the dead area as well as the lack of morphological signs of necrotic cells on hematoxylin and eosin stained sections, like pale or absent nuclei or hypereosinophilia of the cytoplasm suggest that the dominant form of cell death was apoptosis.

The tumor-cell-killing effect of mEHT and the consequent reduction in viable tumor cell count has been already demonstrated in several in vitro experiments using other cell lines [10,12,25,42,44], such as U87-MG and A172 human glioma cells [10], OVCAR-3, SK-OV-3, HeLa, and SNU-17 human ovarian and cervical cancer cells [25], CT26 colorectal carcinoma cells [42] and B16F10 mouse melanoma cells [12]. However, the acute viability-reducing ability of mEHT treatment has not yet been confirmed with imaging techniques, in vivo. Furthermore, mEHT therapy has not been investigated before in any TNBC mouse model, possibly due to the lack of appropriate tools. Our study demonstrated a significant reduction of viable tumor cell numbers in vivo already after two mEHT treatments in monotherapy. The decrease in viable cell count, as detected by IVIS imaging in vivo, was corroborated by the determination of tumor destruction ratio on hematoxylin-eosin-stained sections and hypocellularity by Ki67 immunostaining. The area of tumor destruction was cleaved caspase-3-positive, suggesting apoptotic cell death. We were not able to confirm tumor cell destruction using conventional and less sensitive tumor size measuring methods such as the digital caliper and the ultrasound. The most likely explanation for this deficiency is that the apoptotic/damaged tumor area (detected as TDR on the histology sections) had not yet been removed by the immune-system, during the short follow-up period. This hypothesis has been confirmed in another study by our team [40] with longer follow-up, where tumor cell death also manifested in significant tumor size reduction after 5 mEHT treatments.

Ki67 is a proliferation marker. Elevated expression of Ki67 has been associated with poor prognosis in numerous cancer types, including breast cancer [45,46,47,48]. mEHT-treatment of CT26 mouse colorectal adenocarcinoma allografts reduced Ki67 expression in vivo [4]. In the present study, just two mEHT treatments did not reduce Ki67 mRNA and protein. However, 5 treatments were able to significantly reduce Ki67 protein as assessed by immunohistochemistry [40]. Furthermore, the reduced density of live tumor cells on histological sections also suggests reduced proliferation activity of mEHT-treated tumor cells as also detected by IVIS.

Here we described modulated electro-hyperthermia-induced Hsp70 dynamics. Proper timing of treatments is extremely important in the case of cancer treatment to find the most vulnerable window for optimal treatment effects. Based on our data, the maximized effect of mEHT can be expected with the application of the treatments every other day (at 48-h intervals) as the protective machinery is exhausted by 48-h post-treatment and the tumor tissue damage is still extensive at this time point.

We also demonstrated that the anti-tumor effect of mEHT can be enhanced by blocking the Hsp70-mediated defense mechanisms of tumor cells. Moreover, according to the paper by Gabai et al., Hsp70 inhibitors may act as a double-edged sword as their Hsp70 inhibitory effect in the tumor stroma can also abrogate the tumor-promoting microenvironment [49]. Using Quercetin as a sensitizer to conductive hyperthermia (carried out by a regular water bath) was first described in 1984 by Kim et al. on HeLa cells. The authors interpreted the sensitizing effect of Quercetin on hyperthermia by inhibition of lactate transport [50]. Since then, the synergistic effect of Quercetin with conductive hyperthermia has been demonstrated in leukemic cells [51], melanoma cells [52], and prostate cancer, with synergism interpreted as a result of HSP70 inhibition in all three cases [53]. In this paper, we demonstrated in vitro for the first time, that Quercetin was able to diminish the induction of Hsp70 mRNA upon capacitive hyperthermia (using mEHT technology) in 4T1 cells, and could be used for synergistically potentiating mEHT treatment. The Quercetin concentration (50 μM = 15 μg/mL) used in the in vitro experiments corresponds to patients’ plasma concentrations 1 h after intravenous administration (25–50 mg/kg = 945–1700 mg/m^2^). This is a magnitude higher than steady state plasma concentration (~1 μg/mL) in patients after Quercetin administration [54,55,56]. However, the plasma concentration of Quercetin in humans can cover a very wide range (500 ng/mL–0.1 μg/mL, depending on dosage and way of administration [57]). Our in vitro model served as the proof of principle of using an HSR inhibitor as a sensitizer to modulated electro-hyperthermia. Based on our findings, we hypothesized, that a more specific and more effective inhibitor of the Hsp70 machinery: KRIBB11 could enhance the tumor-killing effect of mEHT even more efficiently. Quercetin is quite pleiotropic in mechanisms of action, and its Hsp70 inhibitory effect is the consequence of the indirect inhibition of Hsf-1 by blocking its hyperphosphorylation and thus its activation [17,18]. Since Hsf-1 is the central regulator of the cellular heat shock response, its more direct and specific blockade could lead to enhanced synergism with mEHT [21]. Therefore, we combined mEHT with the novel direct Hsf-1 inhibitor, KRIBB11. This molecule was introduced in 2011 by Yoon et al. as a highly effective Hsp70 synthesis inhibitor, as it binds directly to Hsf-1 and abolishes its Hsp70-promoting activity very specifically [22,23]. In the past few years, an increasing number of articles have been published about KRIBB11, however, it hasn’t been investigated yet as a sensitizer to hyperthermia. Here we presented for the first time, that KRIBB11 can be combined effectively with hyperthermia treatment, based on its Hsp70-inhibitory effect. The synergism of mEHT with Hsp-70 inhibition should be confirmed and the pharmacokinetics and bioavailability of KRIBB11 should be determined in humans as such data are not yet available. Our results suggest, that inhibition of Hsp70 synergize with modulated electro-hyperthermia and thus could enhance the anti-cancer therapeutic effects of mEHT.

## 4. Materials and Methods

### 4.1. Cell Culture

4T1 cells transfected with firefly luciferase were provided by Judy Lieberman (Lieberman Laboratory, Harvard University, Boston, MA, USA) and grown as adherent culture in Dulbecco’s Modified Essential Medium (DMEM 4.5 g/L glucose with L-glutamine, #12-604F, Lonza A. G., Basel, Switzerland) supplemented with 10% Fetal Bovine Serum (FBS (South America Origin), EU approved, #ECS0180L, Euroclone S.p.A., Pero, Italy), and 10% Penicillin-Streptomycin (Penicillin-Streptomycin Mixture, 10K/10K, #17-602E, Lonza A. G., Basel, Switzerland).

### 4.2. In Vivo Model

4T1 tumor cells transfected with firefly luciferase were grown in cell culture and processed for inoculation in vivo as described before [13,15]. Six-to-eight-week-old female BALB/c mice were raised in the SPF department of the Animal Facility of the Basic Medical Science Center of Semmelweis University with ad libitum access to standard rodent chow and water, under 12 h dark/12 h light cycles. Animals were anesthetized for tumor cell-inoculation with isoflurane (Baxter International Inc., Deerfield, IL, USA) in 4–5% concentration for induction and 1.5–2% to maintain anesthesia with 0.4–0.6 l/min compressed airflow. 1 × 10^6^ 4T1 cells in 50 μL 1:1 Matrigel^®^-PBS (Matrigel^®^ Basement Membrane Matrix, 354234, Corning^®^, NY, USA; Phosphate Buffered Salin without Calcium and Magnesium #17-516F, Lonza A.G., Basel, Switzerland) solution were subcutaneously inoculated by 50 μL Hamilton syringe (Hamilton Company, Reno, NV, USA). Inoculation was made orthotopically into the 4th mammary gland’s fat pad in each mouse [58]. Six days after inoculation tumors were measured with digital caliper and ultrasound and mice were randomized into mEHT and sham-treated groups according to tumor size, age, and weight. Animals (*n* = 7) with the largest, smallest, or twin tumors were excluded from the study. Twenty-four hours after the last treatment mice were euthanized by cervical dislocation. The tumors were resected and cleaned of the surrounding connective tissue, fat, and skin. The condition of the internal organs (bowels, urinary bladder, spleen) and possible adherences between the tumor and muscles were inspected. Tumors were cut in half precisely along their longest diameter, one half was placed in a 4% formaldehyde solution (Molar Chemicals Kft., Halásztelek, Hungary) that was replaced with 70% alcohol after forty-eight hours. Samples were stored for further histological processing for a maximum of 2 weeks. The other half of the tumors were frozen in liquid nitrogen for molecular analysis (RNA isolation, RT-PCR). To investigate the time kinetics of mEHT treatment effects, an experiment was performed the same way as described previously, except tumors were harvested at different time points: 4, 12, 24, 48, and 72 h after the last treatment. Interventions and housing of the animals conform to the Hungarian Laws No. XXVIII/1998 and LXVII/2002 about the protection and welfare of animals, and the directives of the European Union. Our study protocol was approved by the National Scientific Ethical Committee on Animal Experimentation under Nos. PE/EA/633-5/2018 and PE/EA/50-2/2019.

### 4.3. Tumor Size Measurement

Tumor size measurement by ultrasound (Phillips Sonos 5500, Philips, Amsterdam, Netherlands) and digital caliper (Fine Science Tools lnc., Foster City, CA, USA) was performed on the seventh day after inoculation under anesthesia as described previously. Tumors were visualized with ultrasound in two perpendicular planes. Two diameters of the tumor (a, b) were measured and the third diameter was averaged from the depth of the tumor measured in the two planes (c = (c_1_ + c_2_)/2). Three perpendicular diameters (a, b, c) were also measured with a digital caliper. Assuming an ellipsoid form, the volume (*V*) of tumors was calculated based on both the ultrasound and the caliper data by the following formula: *V* = (a × b × c × π)/6

The number of viable tumor cells were measured by in vivo imaging on the third and seventh day after inoculation by the IVIS Lumina System (PerkinElmer Inc., Waltham, MA, USA) [15,59]. The transfected cells constitutively express firefly luciferase which produces bioluminescence after the addition of its substrate. 15 mg/mL D-luciferin (XenoLight D-Luciferin Potassium Salt # 122799, PerkinElmer Inc., Waltham, MA, USA) was administered intra-peritoneally (i.p.) in 150 mg/kg dose. Animals were anesthetized by 100 mg Ketamine/10 mg Xylazine/kg (Calypsol^®^ 50 mg/mL, Richter Gedeon, Budapest, Hungary; CP-Xylazin 2%, Produlab Pharma, Raamsdonksveer, Netherlands) i.p. 3 min after the administration of D-luciferin. The number of emitted photons/second (Total flux) was measured 18, 19, and 20 min after the administration of D-luciferin, and the average was calculated.

### 4.4. In Vivo Treatments

**Figure 11:**
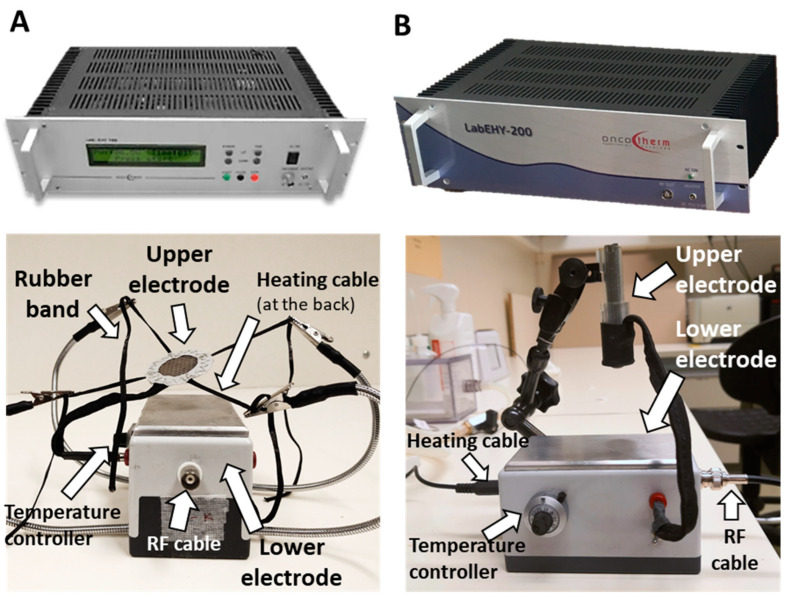
Modulated electro-hyperthermia small animal treatment device. (**A**) LabEHY-100 device and the treating setup: temperature-controlled (heated) lower electrode and tissue electrode (upper electrode). (**B**) LabEHY-200 device and the newly developed treating setup: temperature-controlled (heated) lower electrode and position-adjustable pole electrode (upper electrode).

Animals were treated by modulated electro-hyperthermia (mEHT) under isoflurane anesthesia with the upgraded LabEHY-200 device with improved modulation and a newly designed pole electrode with improved tissue-coupling (Figure 11B) (Oncotherm Ltd., Budaörs, Pest, Hungary). The principle of the treatment is similar to the LabEHY-200 and the previous LabEHY-100 (Figure 11A): The machine generates electromagnetic field by capacitively coupled, amplitude-modulated (AM), 13.56 MHz radiofrequency. Technical improvements include a new tuning method performed by a warbler which is identical to the clinically used method. The new device is connected to a PC by a USB port and the treatment parameters (time, power, modulation) can be adjusted by software with a more user-friendly graphical interface.

**Figure 12:**
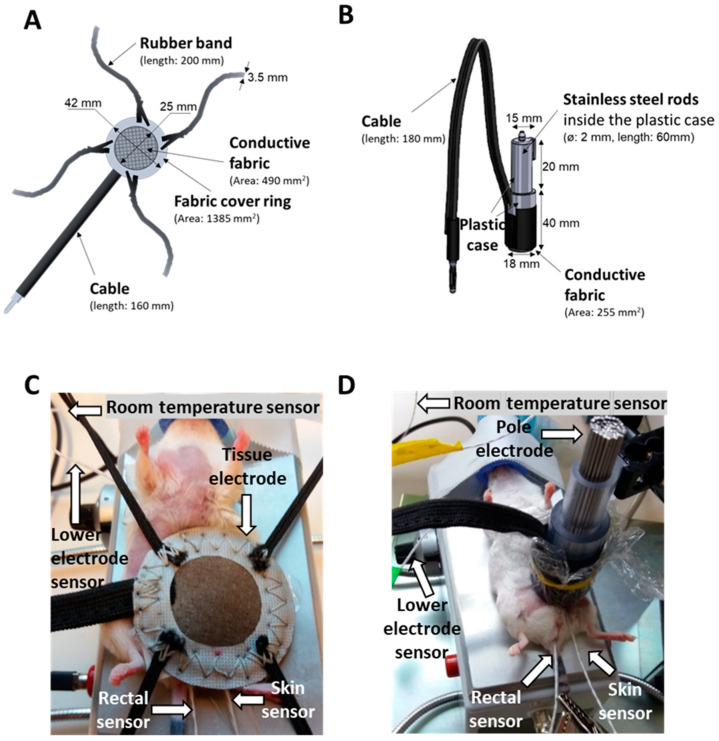
Comparison of the mEHT treatment setups. Schematic diagram and dimensions of the (**A**,**C**) previously used tissue electrode and (**B**,**D**) improved pole electrode. Animal on the lower electrode with (**A**,**C**) the tissue electrode and (**B**,**D**) the pole electrode. The electromagnetic field was established between the lower electrode and the upper electrode positioned on the tumor. The temperature at the four locations is monitored by temperature sensors (rectal, skin, lower electrode, and background).

The previously used tissue electrode was too large (total area: 1385 mm^2^, conductive area: 490 mm^2^), it did not adapt properly to the concave surface of the inguinal region, therefore skin contact and coupling were inappropriate (Figure 12A,C).

The new ergonomic pole electrode adapts to the surface of the tumor and the inguinal region. The size of the electrode was optimized (conductive area: 255 mm^2^; Figure 12B,D).

Before treatments, the inguinal region and the back of the mouse were shaved for better coupling. The electromagnetic field (EMF) around the tumor was generated between the upper and the lower electrode. Mice were laid on the thermo-adjustable lower electrode, connected to the LabEHY-200 with a heating cable and a radio frequency (RF) cable. The temperature of the lower electrode was manually adjusted. The upper electrode was a column-shaped plastic case with ∅: 2 mm stainless steel rods inside, covered with 2.55 cm^2^ silver-plated textile (conductive fabric) to form an easily adapting surface. For preventing hot spot formation and consequent burning, a thin layer of insulator (cellophane) was placed between the electrode and the skin, then the electrode was positioned on the tumor (Figure 12B,D).

Treatments were performed temperature-driven for 30 min with 0.7 ± 0.3 watts on average after a 5-min warmup time. The temperature rise was 1.5 °C/min, corresponding to a SAR of 90 W/kg. The amplitude-modulation (AM) wave used had a fixed 30% depth at the carrier frequency. The frequency spectrum was 1/f (pink noise), where f = 1 Hz–1 kHz. Temperatures were monitored by the four-channel TM-200 thermometer (Oncotherm kft, Budaörs, Hungary). Four temperature-sensors were positioned (1) on the skin above the tumor, (2) in the rectum, (3) on the surface of the lower electrode, and (4) next to the treatment setup (room temperature). Skin temperature was kept at 40 ± 0.5 °C during treatment and rectal temperature was maintained within the physiologic range (37.5 ± 0.5 °C) by adjusting the temperature of the lower electrode. To prevent anesthesia-related hypothermia of the mice, the lower electrode was kept at 37.5 ± 0.5 °C. Treatments were performed at room temperature (25 ± 1 °C). The only difference between treated and sham mice was that the electric field was not turned on in the sham group.

### 4.5. In Vitro Treatments

**Figure 13:**
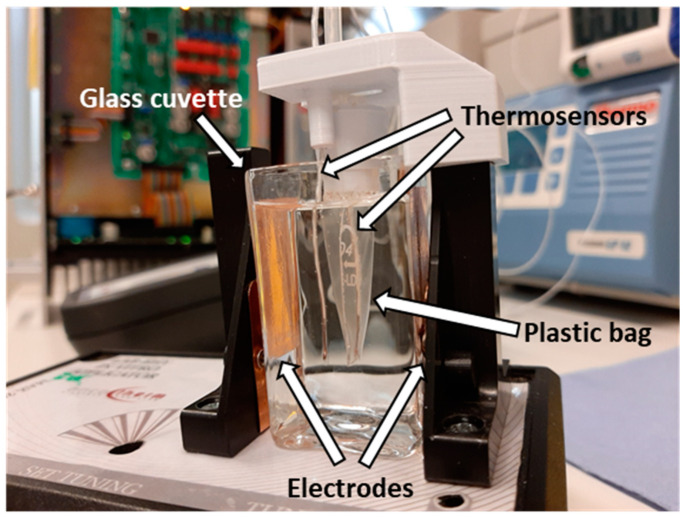
In vitro treatment setup.

Cells were incubated with 50 μM Quercetin, (#Q4951, Sigma-Aldrich Co., St. Louis, MO, USA), 5 μM KRIBB11 (#385570, Sigma-Aldrich Co., St. Louis, MO, USA) or 0.01% DMSO (#D2438, Sigma-Aldrich Co., St. Louis, MO, USA) for 1 h. Next, cells in suspension were transferred into a small plastic bag, optimized for use with the LabEHY 200 in vitro applicator and closed hermetically with a silicone plug. Through the silicone plug, a thermosensor (TM-200 thermometer, Oncotherm kft, Budaörs, Hungary) was inserted into the plastic bag for temperature follow-up during treatment (Figure 13).

The unit containing the cell suspension was placed in a glass cuvette with distilled water, which was inserted between the two electrodes on the LabEHY-200 in vitro applicator (Oncotherm kft, Budaörs, Hungary). Cells were treated for 30 min in a temperature-driven way, for maintaining 42 °C in the cell suspension. An average of 4 ± 1 watt was applied. The amplitude-modulation (AM) was the same as in the in vivo experiments. The temperature rise of the cell suspension was around 2.3 ± 0.8 °C/min, corresponding to a SAR of 138 ± 48 W/kg. Two hours after treatment, 200,000 cells were removed for the viability assay. The remaining cells were lysed with Tri-Reagent (#TR118/200, Molecular Research Center, lnc., Cincinnati, OH, USA) for collecting RNA. Twenty-four hours post-treatment, a resazurin (#R7017, Sigma-Aldrich Co., St. Louis, MO, USA) viability assay was performed.

### 4.6. Histopathology and Immunohistochemistry

Tumor tissues fixed in 10% neutral-buffered formalin were dehydrated and embedded in paraffin. Serial sections (2.5 µm) were cut, mounted on silanized glass slides, and kept in a thermostat at 65 °C for 1 h. Sections were dewaxed and rehydrated for hematoxylin-eosin (H&E) staining and immunohistochemistry (IHC). For antigen retrieval, heating for 20 min in Tris-EDTA (TE) buffer pH 9.0 (0.1 M Tris base and 0.01 M EDTA) using an Avair electric pressure cooker (ELLA 6 LUX(D6K2A), Bitalon Kft, Pécs, Hungary) was performed, followed by a 20-min cooling with an open lid. Endogenous peroxidases were blocked for 15 min using 3% H_2_O_2_ in methanol. The non-specific proteins were blocked also for 15 min in 3% bovine serum albumin (BSA, #82-100-6, Millipore, Kankakee, Illinois, USA) diluted in 0.1 M Tris-buffered saline (TBS, pH7.4) containing 0.01% sodium azide. The sections were incubated with the primary antibodies diluted in 1% BSA/TBS + TWEEN (TBST, pH 7.4) (Table 1.) overnight in a humidity chamber. Peroxidase-conjugated anti-rabbit & anti-mouse IgGs (HISTOLS-MR-T, micropolymer -30011.500T, Histopathology Ltd., Pécs, Hungary) were used for 40 min incubations and the enzyme activity was revealed in 3, 3’-diaminobenzidine (DAB) chromogen/hydrogen peroxide kit (DAB Quanto-TA-060-QHDX-Thermo Fischer Scientific, Waltham, MA, USA) under microscopic control. All incubations were at room temperature with the samples washed between incubations in TBST buffer for 2 × 5 min.

Slides were digitalized by Pannoramic Digital Slide Scanner (3DHISTECH Ltd., Budapest, Hungary) and the reactions were evaluated with the CaseViewer image-analysis software (3DHISTECH Ltd., Budapest, Hungary). The tumor area was annotated digitally and the area with positive immunoreaction was masked by setting the intensity, color, and saturation in the annotated area on each staining with the QuantCenter module of CaseViewer. The ratio of the masked area to the annotated area (relative mask area = rMA) was used to estimate the expression of the target molecule. TDR (%) was calculated on H&E- and cC3-stained slides dividing the whole tumor area with the pale, damaged (Figure 4C), or cC3-positive area. Matrigel was distinguished from the damaged area as the unstructured, homogeneous, cell-poor, eosinophilic area on H&E (Figure 4C) and the same but aspecific pale brown on cC3 staining (Figure 4B). rMA of Hsp70 and Ki67 was measured in the intact tumor area, which was determined previously on the cC3 stainings as cC3 negative areas (see Table 1 for the antibodies used). Hsp70 was evaluated regardless of cellular location (HistoQuant module). In the case of Ki67, nuclei were counted and the number of positive nuclei and the number of positive and negative (all nuclei) in the live area were counted.

### 4.7. RNA Isolation and mRNA RT-PCR

RNA isolation was performed with TRI reagent (Molecular Research Center lnc., Cincinnati, OH, USA) according to the manufacturer’s instructions. Reverse transcription of isolated RNA was performed by High-Capacity cDNA Reverse Transcription Kit (Applied Biosystems, Foster City, CA, USA). Amplified cDNA was used as a template for RT-PCR. For the detection of mRNAs in samples, SYBER Green-based RT-PCR was applied, performed with SsoAdvanced™ Universal SYBER^®^ Green Supermix and CFX96 Touch Real-Time PCR Detection System (Bio Rad, Hercules, CA, USA). Expressions were normalized to GAPDH. The primers used in our study are shown in Table 2.

### 4.8. Statistical Analysis

Statistical analysis was performed using GraphPad Prism software (v.6.01; GraphPad Software, Inc., La Jolla, CA, USA). Treated and sham groups were compared with the unpaired Mann–Whitney nonparametric test. In the follow-up examinations, two-way ANOVA with Bonferroni correction was performed. Differences were considered statistically significant as * *p* < 0.05, ** *p* < 0.01, *** *p* < 0.001, **** *p* < 0.0001. Data are presented as mean ± SEM values.

## 5. Conclusions

An improved rodent mEHT device and a novel ergonomic pole electrode allowed us to successfully reduce viable tumor cells in a triple-negative breast cancer isograft model with mEHT monotherapy. As short-term effects, observed after 1–2 mEHT treatments, viable tumor cell count decreased and large, damaged areas were observed in mEHT-treated tumors which were positive for cleaved caspase-3, confirming apoptotic cell death. The area surrounding the damaged tumor tissue strongly expressed Hsp70, supporting a central role for cell stress in the observed mEHT effect culminating in caspase-dependent apoptosis. This effect was most prominent twenty-four hours after treatment when Hsp70 expression was already diminished. Inhibition of the heat-shock factor-1 by Quercetin or KRIBB11 could synergistically enhance mEHT-induced tumor destruction to verify a major role in the loss of cell protection against stress. Furthermore, our data suggest that repeating mEHT treatments after a 48-h interval may result in the most effective tumor destruction, at least in this isograft model.

These preclinical studies may call attention to the potential benefits of mEHT in clinical settings to be exploited for combination therapies with the available regiments, e.g., in neoadjuvant therapy of locally invasive breast cancer (e.g., TNBC) before surgery.

## Figures and Tables

**Table 1:** Antibodies and conditions used for immunohistochemistry and immunofluorescence.

Antigen	Type	Reference No.	Dilution	Vendor ^1^
Cleaved caspase-3	Rabbit, pAb	#9664	1:300	Cell Signaling
Hsp70	Rabbit, pAb	#4872	1:200	Cell Signaling
Ki67	Rabbit, pAb	#RM-9106	1:400	Thermo

^1^ Vendor specification: Cell Signaling (Danvers, MA, USA), Thermo (Waltham, MA, USA).

**Table 2:** Primers used for RT-PCR.

Gene Symbol	Gene Name	Primer Pairs
HSPA1A	heat shock protein 70 [Mus musculus]	Fwd: ATGGACAAGGCGCAGATCC Rev: CTCCGACTTGTCCCCCAT
GAPDH	glyceraldehyde-dehydrogenase [Mus musculus]	Fwd: CCAGAATGAGGATCCCAGAA Rev: ACCACCTGAAACATGCAACA
MKI67	Ki67 [Mus musculus]	Fwd: GAAGTCAAAGAGCAAGAGGCAA Rev: TTCTGTTGGCTTGCTTCCATC
18S	18S [Mus musculus]	Fwd: CTCAACACGGGAAACCTCAC Rev: CGCTCCACCAACTAAGAACG

## Abbreviations

TNBC	Triple-negative breast cancer
HER2	Human epidermal growth factor receptor 2
mEHT	Modulated electro-hyperthermia
Hsp70	Heat shock protein 70
DAMP	Damage-associated molecular pattern
MMTV+	Mouse mammary tumor virus positive
cC3	Cleaved caspase-3
RT-qPCR	Real-time quantitative polymerase chain reaction
IVIS	In vivo imaging system
RF	Radio frequency
H&E	Hematoxylin and eosin
IHC	Immunohistochemistry
TDR	Tissue damage ratio
